# Mitigating Under-Sampling Artifacts in 3D Photoacoustic Imaging Using Res-UNet Based on Digital Breast Phantom

**DOI:** 10.3390/s23156970

**Published:** 2023-08-05

**Authors:** Haoming Huo, Handi Deng, Jianpan Gao, Hanqing Duan, Cheng Ma

**Affiliations:** 1 Beijing National Research Center for Information Science and Technology, Department of Electronic Engineering, Tsinghua University, Beijing 100084, China; 2 Institute for Precision Healthcare, Tsinghua University, Beijing 100084, China; 3 Institute for Intelligent Healthcare, Tsinghua University, Beijing 100084, China

**Keywords:** photoacoustic imaging, breast imaging, deep learning, under-sampling, image reconstruction

## Abstract

In recent years, photoacoustic (PA) imaging has rapidly grown as a non-invasive screening technique for breast cancer detection using three-dimensional (3D) hemispherical arrays due to their large field of view. However, the development of breast imaging systems is hindered by a lack of patients and ground truth samples, as well as under-sampling problems caused by high costs. Most research related to solving these problems in the PA field were based on 2D transducer arrays or simple regular shape phantoms for 3D transducer arrays or images from other modalities. Therefore, we demonstrate an effective method for removing under-sampling artifacts based on deep neural network (DNN) to reconstruct high-quality PA images using numerical digital breast simulations. We constructed 3D digital breast phantoms based on human anatomical structures and physical properties, which were then subjected to 3D Monte-Carlo and K-wave acoustic simulations to mimic acoustic propagation for hemispherical transducer arrays. Finally, we applied a 3D delay-and-sum reconstruction algorithm and a Res-UNet network to achieve higher resolution on sparsely-sampled data. Our results indicate that when using a 757 nm laser with uniform intensity distribution illuminated on a numerical digital breast, the imaging depth can reach 3 cm with 0.25 mm spatial resolution. In addition, the proposed DNN can significantly enhance image quality by up to 78.4%, as measured by MS-SSIM, and reduce background artifacts by up to 19.0%, as measured by PSNR, even at an under-sampling ratio of 10%. The post-processing time for these improvements is only 0.6 s. This paper suggests a new 3D real time DNN method addressing the sparse sampling problem based on numerical digital breast simulations, this approach can also be applied to clinical data and accelerate the development of 3D photoacoustic hemispherical transducer arrays for early breast cancer diagnosis.

## 1. Introduction

Breast cancer is a prevalent global health issue with over 2.3 million new cases diagnosed in 2020, leading to 685,000 deaths [1,2]. Early detection is crucial for reducing mortality rates, but current screening methods have limitations. For instance, mammography poses risks to human health and has a high false positive rate in dense breasts [3,4]. Ultrasound is a low-cost option but is less effective in deeper breast tissue [5,6]. MRI has excellent resolution and sensitivity, but its high cost and time-consuming nature make it unsuitable for early diagnosis [7,8,9]. As a result, photoacoustic (PA) imaging has garnered significant attention in breast cancer screening and diagnosis due to its ability to overcome many limitations associated with other methods [10,11,12].

PA imaging is a non-invasive imaging technique that uses a pulsed laser to illuminate biological tissue, such as vasculature, lipid and fat, where the scattered photons are absorbed by chromophores in the tissue, leading to rapid thermal expansion and the generation of ultrasonic waves [13]. Ultrasonic transducers then detect these sound waves and reconstruct images based on light absorption contrast. Compared with traditional optical imaging methods, PA imaging allows for deeper tissue imaging up to multiple centimeters due to weak acoustic scattering [14].

Recent efforts in PA imaging have focused on enhancing its high-speed imaging capability in both 2D and 3D [15,16]. Achieving higher temporal resolution has involved the use of sparse sampling, which may lead to image quality degradation and various reconstruction artifacts [17,18,19,20]. During image reconstruction, regularization terms, such as total variation (TV), can be added to mitigate reconstruction artifacts. However, such a technique is not suitable for the recovery of thin vessels, and more sophisticated regulation techniques are needed to recover anisotropic structures [19].

Deep learning methods have revolutionized the reconstruction and processing of biomedical images [21,22,23]. The utilization of U-Net-based convolutional neural networks (CNNs) becomes popular in addressing various biomedical imaging issues due to CNNs’ capability to integrate multiple-level features and to be adapted to various imaging modalities [24]. In the field of PA imaging, U-Net was successfully applied to the removal of artifacts caused by limited sampling data [25,26], vessel segmentation [27], and reconstruction of initial pressure images [28]. To be more specific, U-Net-based CNNs show outstanding performances to restore image details by removing artifacts generated when the spatial sampling is below the Nyquist criterion. Antholzer et al. [25] used U-Net to process PA reconstructed images collected from 30 sparsely distributed transducers, and the results show a faster imaging speed and richer image details compared with filtered back projection (FBP). Guan et al. proposed an improved network based on U-Net, named FD-UNet, to post-process reconstructed mouse brain vasculature images using time reversal [26]. Deng et al. applied SE-UNet with an attention mechanism to remove noise and artifacts stemming from under-sampling [29]. Choi et al. applied an improved version of U-Net named 3D-pUnet to address the limited view artifacts caused by clustered-sampling [30]. However, none of the existing methods were directly applied to 3D PA images for human breast vasculature. Utilizing post-processed 2D images that are sliced to reconstruct a 3D image can lead to feature discontinuity.

**Figure 1:**
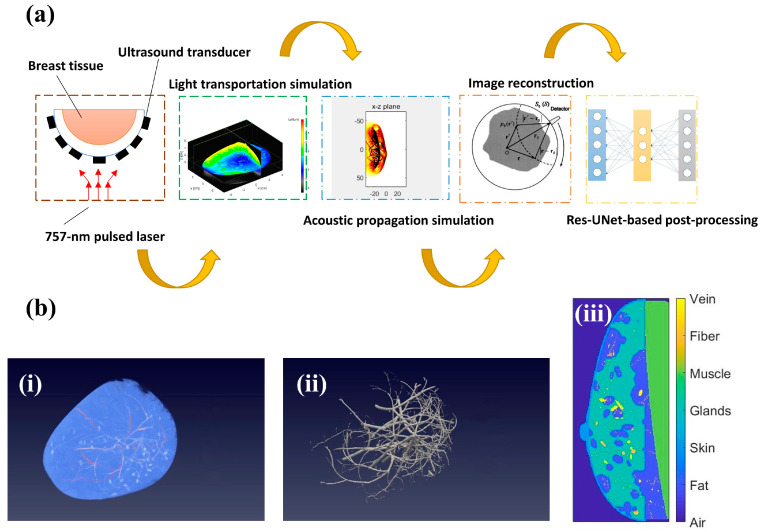
(**a**) Pipeline utilizing Res-UNet model to solve under-sampling problem based on PA numerical breast phantom; (**b**) (i) Overview of numerical breast phantom; (ii) Extracted blood vessel structure; (iii) Numerical breast phantom components in sagittal plane.

In this study, we aim to apply U-Net-based postprocessing directly to 3D PA images to remove under-sampling artifacts. In order to do so, we relied on simulated breast imaging data. Researchers have conducted numerical studies to simulate the process of 3D PA imaging in human breasts, using a combination of simple objects to represent background soft tissue [31]. Additionally, tumor models derived from mice studies were explored to simulate 3D breast tumor structures, including skin, vessels, fat, and fibroglandular tissue [32]. To further improve the accuracy of breast structure representation, contrast-enhanced MRI breast scanning data were segmented and used as the PA source [33]. A recently developed software called VICTRE 1.0 breast phantom can serve as a realistic 3D model for breast structure [34]. The models generated by the software have very similar structure and parameters to real breast tissues. Here, we proposed a pipeline that demonstrates the crucial role of utilizing Res-UNet-based networks to effectively improve image quality in the case of spatial under sampling, based on 3D PA images generated from numerical simulations. The whole scheme is depicted in Figure 1a, which consists of digital breast generation, light transportation simulation, acoustic propagation simulation, image reconstruction and Res-UNet-based post-processing.

## 2. Materials and Methods

### 2.1. Volumetric Image Acquisition and Preprocessing

Figure 1b(i) shows the overview of the VICTRE **1.0** breast model. In order to better represent the breast’s position in a clinical setting, we adjust the parameters of the breast model to simulate a prone position, and the laser illuminates the coronal plane vertically. A hemispherical-array-based PA imaging system is simulated in this study, and we set the breast density to heterogeneously dense (0.339 GVF) [3]. Figure 1b(ii) shows the extracted blood vessel structure. Once the basic breast parameters are fixed, 140 digital breast models were generated automatically based on pre-determined statistical values with different shapes, including air, fat, skin, glands, muscle, fiber and vein, as shown in Figure 1b(iii) [35]. Compared to other imaging modalities, PA imaging is advantageous in achieving non-invasive imaging of blood vessels and blood oxygenation, due to the high absorption of oxygenated and deoxygenated hemoglobin [36].

In our study, we used MCmatlab [37] as the simulation platform for light transport in various tissue. The simulation zone was defined as a rectangular cuboid with a uniform division into cubic voxels, and the distance of each voxel was defined as 0.25 mm. The computation volume was set to be 359 × 287 × 153 (voxels). Each voxel was assigned a specific medium or tissue type based on its absorption coefficient *µ*_a_, scattering coefficient *µ*_s_, and the Henyey–Greenstein scattering anisotropy factor *g* at the given optical wavelength. The input light beam was simulated by launching photon packets at the tissue’s surface, and the 3D optical energy deposition within the concerned volume was calculated using a MC model. Near-infrared (NIR) light at a wavelength of 757 nm was used for PA signal excitation due to its low attenuation inside breast tissues [38]. The light source was placed outside the digital breast to provide a uniform illumination with a radius of 1.2 cm. The optical parameters for the optical simulation are listed in Table 1 [39,40].

**Figure 2:**
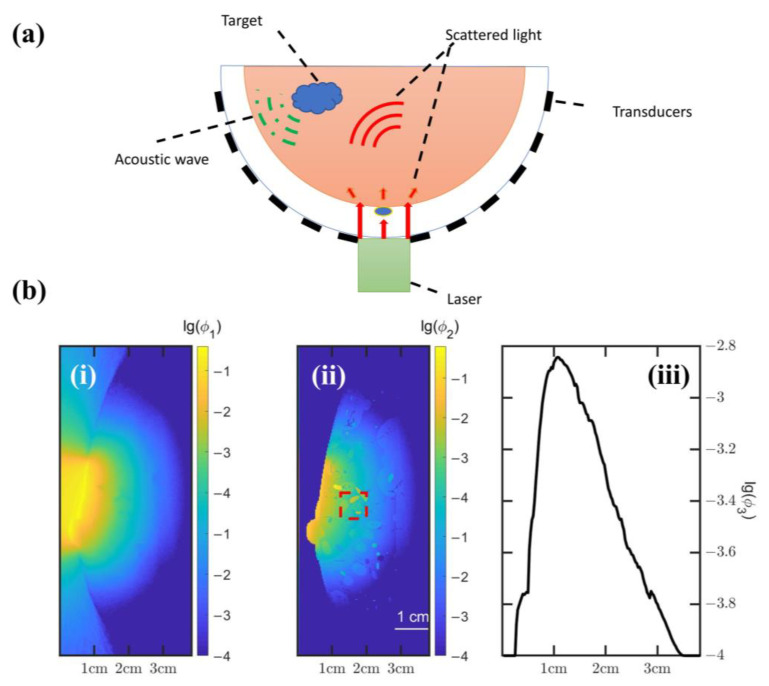
(**a**) The diagram of photoacoustic imaging simulation using a hemispherical transducer array; (**b**) (i) Cross-sectional view of the light intensity distribution, denoted by *ϕ*_1_; (ii) Cross-sectional view of light absorption distribution, denoted by *ϕ*_2_; (iii) Average energy deposition at different depths, denoted by *ϕ*_3_.

In our acoustic simulation, we used a hemispherical array to take advantage of its 2π solid-angle coverage, which partially alleviates the limited-view problem. First, we simulated an ultrasound detector array consisting of 5120 transducer elements uniformly distributed on the hemisphere, having an overall radius of 4.8 cm. Due to the fact that two adjacent locations on the regular grid provide very similar information, random distribution of transducer elements will reduce the coherence of measured signals and yield better signal quality [19]. However, taking into account the limitations of simplicity and manufacture cost, equidistant sampling is adopted as the basic sampling strategy. The central frequency of each element is 2.5 MHz, and the—6 dB bandwidth is 70%. The configuration can be adjusted to simulate under-sampling at various degrees. The diagram of our imaging setup is presented in Figure 2a. In our simulations, the gap between the transducer array and the breast was filled with water to reduce acoustic and optical reflections on the skin.

The depth of photoacoustic imaging is limited due to the significant light absorption and scattering during light propagation in tissue. Figure 2b(i,ii) shows notable attenuation of the light intensity as the depth of penetration increases. Figure 2b(iii) shows the average energy deposition at different depths. The penetration depth of current simulation can reach 3 cm. The red dashed box in Figure 2b(ii) represents the cross-sectional feature of an arterial vessel.

We used the K-wave MATLAB toolbox [41] for the acoustic propagation simulation. Based on the 3D MC model, the initial PA pressure was generated first using the optical simulation described above. For simplicity, we assumed that the breast was acoustically uniform, and the speed of sound was 1500 m/s with no attenuation. The distance for each voxel was defined as 0.25 mm as well. The transducer elements used for detecting PA signals were curved along the radial direction of the hemispherical shell. Each transducer element occupied one voxel space. To achieve efficient image reconstruction, a 3D delay-and-sum algorithm with GPU acceleration was used to form a 3D single-wavelength image. The images reconstructed using all 5120 transducer elements served as the ground truth to train the deep-learning module. In our experiment, data were collected with under-sampling rates of 5%, 6%, 7%, 8%, 9% and 10%, corresponding to 256, 310, 360, 410, 460, and 512 elements. The active elements were selected from the fully sampled data with equidistant sampling. Subsequently, PA images were reconstructed, and 140 pairs of images comprised of sparsely-reconstructed PA image and ground truth image for each under-sampling ratio were grouped into training, validation, and testing sets with a ratio of 0.8:0.1:0.1. To accelerate computation, we cropped each 3D image into 310 × 310 × 128 voxels around the region of interest (ROI). We reduced the number of voxels in the coronal plane by two times with bicubic down sampling to compress irrelevant information. In our study, we used patch-wise segmented images instead original images as input. The volumetric images were augmented by random cropping into a size of 96 × 96 × 96 followed by random flipping around the horizontal and vertical axes to expand the training set. In terms of the validation and testing sets, we cropped each sample with equal distance in the coronal, sagittal and transverse planes, into the size of 96 × 96 × 96 overlapping patches. The reason why we use patch-wise processing is to enlarge dataset scale, enhance generalization and reduce memory consumption. The corresponding outputs of the network were concatenated in the initial order. A Gaussian filter was applied before image stitching to mitigate edge effects in the final output [42].

### 2.2. Res-UNet Architecture

The Res-UNet architecture was proposed in [43] as an approach to address the low discriminative ability issue encountered during segmentation tasks involving small features. We customized it into a pix2pix architecture for the image-to-image problem. It incorporates residual blocks into the contracting and symmetric expanding paths of the U-Net architecture, effectively mitigating the gradient vanishing problem that arises when the network extends deeper. Additionally, this strategy allows for significant deepening of the neural network, leading to further improvements in performance [44]. The Res-UNet architecture not only extracts comprehensive context information from the input sparsely-reconstructed PA images, but also infers the initial pressure distribution from a symmetric-expanding path. Additionally, to refine the original U-Net, a skip connection is included between the input sparse reconstructed PA image and the output initial pressure distribution via element-wise summation, allowing the network to learn quickly and recover the full spatial resolution.

The Res-UNet architecture defines a basic module as a composite function of three consecutive operations: a 3 × 3 × 3 convolution (Conv), batch normalization (BN), and leaky rectified linear unit (LeakyReLu). To enable the Res-UNet to efficiently learn local and global features over different spatial scales, each contracting unit consists of two basic modules and a residual block, with the spatial dimensions of feature maps repeatedly reduced via a 2 × 2 × 2 max pooling operator. The residual block is composed of a 3 × 3 × 3 convolution and added to the output of each contracting unit. In the expanding path, each expanding unit comprises a 2 × 2 × 2 transposed convolution operator followed by two basic modules and a residual block. Another basic module is introduced in the final layer, followed by a 1 × 1 × 1 convolution to compress channel information.

We designed the loss function as a combination of mean absolute error (MAE) with multi-scale structural similarity index metric (MS-SSIM). We chose MAE instead of mean square error (MSE) because MSE yields unsatisfactory outcomes in tasks involving image-to-image translation [45]. MS-SSIM is a comprehensive metric that quantifies the similarities between two images across various spatial scales, taking into account contrast, luminance, and structure. It achieves this by computing similarities using a local pixel neighborhood and obtaining a global value by averaging the neighborhood values, which is superior to signal-to-noise-ratio (PSNR) and standard SSIM for 3D image measurement [46]. The loss function for our 3D Res-UNet is defined as follows:

Loss = 0.3 × L_1_ + 0.7 × (1 − MS-SSIM) (1)

The DNN utilized in this study was implemented using PyTorch 1.10.1 [47], an open-source platform for deep learning that is compatible with Python 3.8. The training and evaluation of the models were conducted on a system consisting of an NVIDIA GeForce RTX 3090 and an Intel^®^-Core™ i9-10900X CPU. To initialize all trainable parameters, the He normal initialization method [48] was employed. The hyper-parameters, including the coefficients of the loss function, were fine-tuned through a grid search approach. During the training process, the Adam optimizer [49] was utilized with a learning rate of 0.0025, weight decay of 0.001, and a batch size of 3. Early stopping was also used, and the training was performed for 300 epochs.

## 3. Results

**Figure 3:**
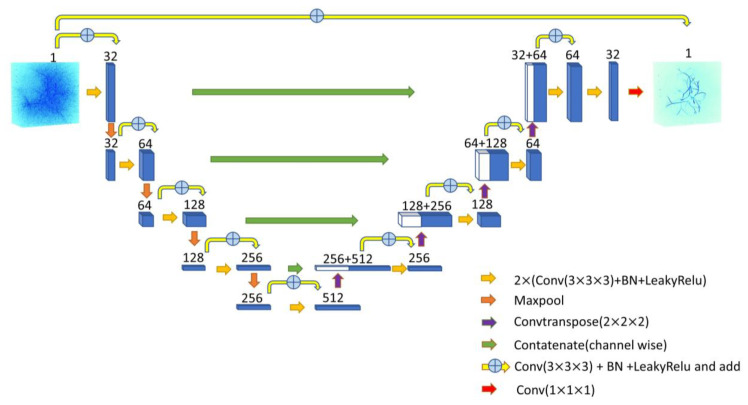
Visual representation of customized 3D Res-UNet architecture.

In Figure 3, we present the results of our 3D Res-UNet framework, where 3D dense reconstructed PA images were reconstructed from the data collected by the 5120-element array, while sparsely-sampled images reconstructed from 512 transducer elements (10% sparsity ratio) were used as the input. The reconstruction time for the fully sampled image was 153 s, while for the sparsely-sampled image it was 117 s, demonstrating the efficiency of our method. The reason why the reduction of time is not proportional to the reduction of sampling points is because the process of utilizing a low-pass filter to enhance signal quality, computed by CPU, took a significant amount of time. We display the reconstructed PA maximum amplitude projection (MAP) images on the coronal and sagittal planes for numerical breast phantoms. It is worth noting that the sparsely-sampled images were used as the input to the network.

**Figure 4:**
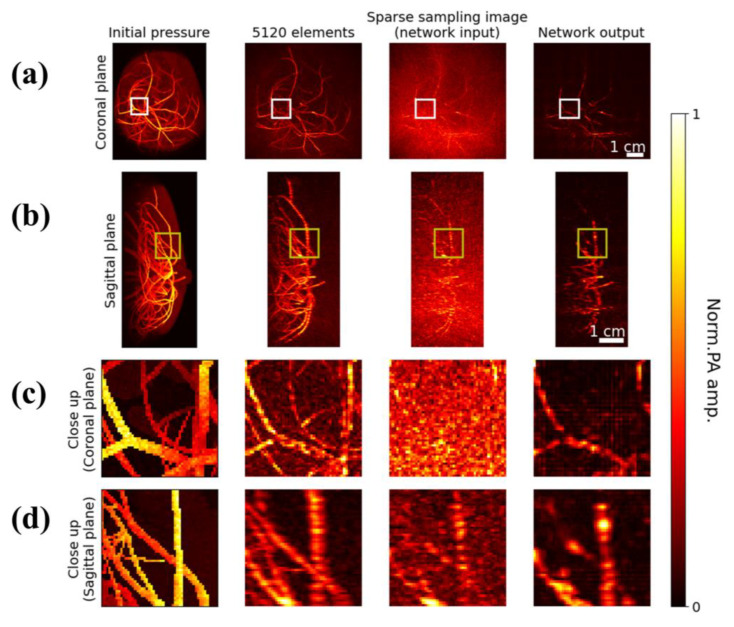
Performance of 3D deep learning network. First column: numerical breast phantom (initial pressure distributions); second column: dense reconstruction results; third column: sparse reconstruction results (under-sampling ratio 10%); fourth column: DNN results (same under-sampling ratio as the third column). (**a**) MAPs in the coronal plane. (**b**) MAPs in the sagittal plane. (**c**) Close-up views of the regions outlined by the white bounding boxes in (**a**). (**d**) Close-up views of the regions outlined by the yellow bounding boxes in (**b**).

Compared to the initial pressure distributions in the digital phantom, the reconstructed blood vessels exhibit slight distortions, partly because we assumed that the tissue is acoustically homogeneous during image reconstruction. Nevertheless, the vasculature features are clearly visible in reconstructed images, as depicted in Figure 4a. Some smaller features located deeper within the tissue displayed in Figure 4b are missing in the reconstructed images. The 3D Res-UNet framework effectively preserved the 3D structural information. The post-processing time for each sample is 0.6 s. It yielded artefact-reduced reconstruction results with errors primarily located within areas having dense blood vessels. The diameters of the vessels were faithfully recovered as well. The white boxes in Figure 4a and the yellow boxes in Figure 4b indicate that the PA images with sparse-sampling showed discontinuous vessel features, compared to the dense-sampling and DNN-processed images. The comparison revealed a remarkable suppression of the artifacts by the DNN, resulting in better resolution of adjacent blood vessels that were obscured in the original sparse-sampling image. However, a few structures were not properly recovered by the network, especially for the vessels located far from the illuminated area, due to a lack of sufficient SNR. To highlight the differences among the images, we show the enlarged images corresponding to the areas in the bounding boxes in Figure 4c,d.

The quality of the reconstructed PA images is directly determined by the number of transducers used for data acquisition. To verify the performance of Res-UNet capability, in addition, we tested our model using PA data with down-sampling ratios ranging from 5% to 10%. Specifically, the number of transducer elements was set to 256, 310, 360, 410, 460, and 512; these elements were arranged equidistantly on the hemisphere as well.

**Figure 5:**
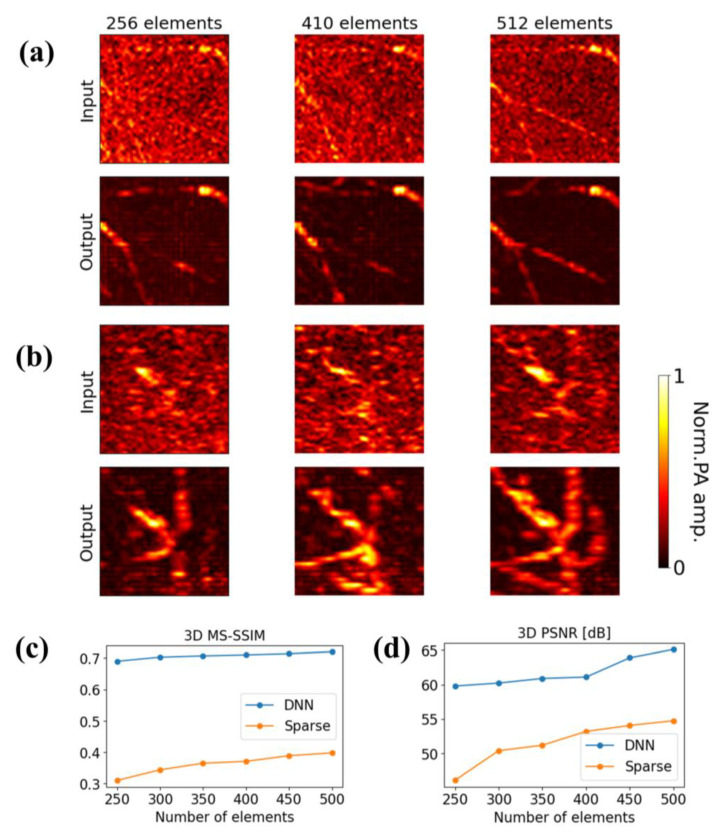
Performance of 3D deep learning network for data with different degrees of sparsity. (**a**) MAPs of ROI in coronal plane and (**b**) MAPs of ROI in sagittal plane on sparse reconstruction results and corresponding DNN outputs for 256, 410 and 512 transducer arrays. First row: sparse reconstruction results. Second row: corresponding DNN outputs. (**c**) 3D MS-SSIM and (**d**) 3D PSNR evaluation metrics for transducer array numbers of 256, 310, 360, 410, 460, and 512.

Representative reconstruction results and corresponding DNN outputs for 256, 410 and 512 transducers in the coronal and sagittal plane are shown in Figure 5a,b, respectively. The superficial vascular structures and connectivity become more apparent with an increase in sparsity ratio, as well as edge smoothness. For a transducer array of 256 elements, the vascular features exhibit significant recovery, while the background artifact is noticeable even though the vasculature shows speckle patterns on the MIP from the coronal plane. The Gaussian-shaped artifact in the sparsely-reconstructed PA images is caused by the combination of insufficient sampling frequency and limited-view problem. As the under-sampling ratio increases, the vascular pattern becomes continuous, and the DNN images become similar to dense images.

In addition, the 3D peak signal-to-noise ratio (PSNR) and 3D multiscale structural similarity (MS-SSIM) between the DNN or sparse images and the dense images were calculated based on the transducer array numbers of 256, 310, 360, 410, 460, and 512, as shown in Figure 5c,d. Both PSNR and MS-SSIM increase with an increase in the number of transducer arrays. Despite the fact that the training and testing sets are from different domains and have different distributions, the lower the under-sampling ratio, the greater the improvement in the model’s performance on the input. At a transducer array number of 256, a PSNR value of 59.87 dB and MS-SSIM of 0.693 were achieved, while the corresponding metrics for the sparse images were 46.1 dB and 0.312, respectively. At a transducer array number of 512, corresponding to an under-sampling ratio of 10%, a PSNR value of 65.1 dB and MS-SSIM of 0.71 was achieved, while the corresponding metrics for the sparse images were 54.7 dB and 0.398, respectively. The reason why the PSNR and MS-SSIM values are not super high is due to the low percentage of vasculature present in the 3D image. However, it is important to note that when the structure has a high sparsity of 10% and more, it cannot be recovered by DNN.

## 4. Discussion

Sparse sampling data offers several advantages, including accelerated image reconstruction, reduced complexity of PA imaging systems, and lower overall costs. However, there are inherent trade-offs among spatial resolution, penetration depth, and imaging speed, which often result in sub-optimal reconstruction outcomes and limit the widespread adoption of this technique in clinical applications. As a result, we developed a relatively fast and efficient image post-processing approach based on a DNN model to remove under-sampling artifacts in PA breast imaging. Our training, validation and testing datasets were obtained through 3D PA simulation using digital breast phantoms that conform to human anatomical structures and physical properties. The simulation process also took into consideration the illumination and ultrasound detection configurations, aiming to ensure similarity to practical applications. Simulation results show that the illumination depth can reach up to 3 cm, which is consistent with the clinical results.

Despite the fact that little distortion appears due to the assumption that the medium is acoustically homogeneous during reconstruction, it does not affect the performance of DNN. The heterogeneous speed of sound distribution can be further explored in the future. The satisfactory network performance with sparsely-sampled data is partly attributed to the good quality of the training images. The method can effectively enhance structural visibility and reduce the speckle-like artifacts as well as the image background artifacts with a post-processing time of 0.6 s for each image. Our results show that the application of DNN can significantly improve image quality by 78.4% measured by MS-SSIM (>0.71) and reduce background artifacts by up to 19.0% measured by PSNR (>65.1 dB) at the under-sampling ratio of 10% (512 transducer array).

However, many physical configurations of the hemispherical array need to be addressed, and applying the above DNN to clinical applications still presents significant challenges. The reconstruction results and subsequent model performances are highly influenced by the time series signal we collected. Lan et al. further validates the benefits of using random masking strategy based on a ring shaped array to achieve higher reconstruction performance in an extreme sparse scenario [50]. The specific transducer array arrangement needs further exploration. In addition, even though the digital breast phantom we adopted has realistic physical properties and distribution, differences between clinical data and simulated data are unavoidable. For example, the quality of clinical data highly relies on various factors such as illumination conditions, the position of the target, and all kinds of noises. Furthermore, the published tissue physical attributes were primarily measured from exercised samples, which may vary greatly from in vivo tissues, and from person to person. These differences are often referred to as domain gap. Additionally, many potentially undefined features may appear in clinical data. These factors can lead to failure of the model even if validated with in silico data. Simple fine-tuning technique is not adequate by the lack of real paired “fully sampled data” (ground truth). Ben et al. delved deeper into the reasons why networks trained on simulated data using conventional methods are unlikely to exhibit generalized performance on real tissue [51]. Domain adaptation is a sub case of transfer learning developed in the field of computer vision [52,53,54], aiming to leverage the related source domain to learn unseen features in the target domain. To be more specific, unsupervised learning techniques can be deployed for the above domain adaptation task without the need of paired images. Ciara, etc. [55] applied a GAN-based structure to reduce the domain gap between synthetic PA images and real PA images, which shows that feature spaces from different domains can be closely aligned. Although the model trained on simulated data can effectively reduce the generation of artifacts, the output still exhibits a relatively large variance when compared to the ground truth images. However, utilizing the Res-UNet model trained on simulated data remains potentially effective in addressing the under-sampling problem in real clinical data. Another approach to tackle the problem is meta-learning, which designs new algorithms that adjust to learn new features constantly while retaining prior knowledge [56]. The goal is to construct a model capable of continued learning from newly available features even after its deployment. The integration of the above-mentioned methods will facilitate the adoption of DNN in the field of 3D PA imaging.

Apart from domain adaption problem, which may cause the degradation of DNN performance on in vivo data, the reconstructed 3D PA images dataset we generated to train in the study were insufficient, additionally when compared to traditional computer vision tasks. Due to the limited number of in-house datasets, the feature patterns that DNN learns may not be adequate for good generalization on another type of digital breast phantoms. Furthermore, these models still have limitations in accurately representing the diverse structure and function of blood vessels. Nevertheless, it is our firm belief that such problem will be solved eventually with the collective efforts of everyone in PA community. In the aid of other modules, extra information from other dimensions can be added to enlarge the source domain and enhance DNN capability.

## 5. Conclusions

In this paper, we presented a real time method to remove under-sampling artifacts in 3D PACT breast imaging based on a Res-UNet model. We found that the use of 3D convolutions can efficiently extract spatial information and restore target features in PA images. We used numerical breast phantoms with shapes and physical parameters similar to realistic breasts to generate PA signals. The proposed method can facilitate the development of more efficient data acquisition method and reduce equipment cost, while accelerating the image reconstruction process. More research needs to be conducted by transferring the trained model on digital breast phantoms to real clinical data. It is worth mentioning that previous studies have demonstrated that integrating neural networks into iterative reconstruction algorithms can yield better results compared to using a U-Net-based network alone; using more advanced network architectures in conjunction with iterative approaches can offer potential image quality enhancement on under-sampling data.

## Figures and Tables

**Table 1:** Optical parameters of various materials/tissue types used in the simulation.

Tissue/Material Type	*μ_a_*/cm^−1^	*μ_s_*/cm^−1^	*g*
Air	1 × 10^−12^	1 × 10^−12^	1
Fat	0.62	73	0.98
Skin	0.48	167	0.9
Glands	0.36	112	0.96
Muscle	0.52	73.6	0.93
Fiber	0.13	115	0.13
Vein	4	71.4	0.9

## Data Availability

The code and data are available upon request.
